# The effect of fruit pomace addition on the color, texture and sensory properties of gluten-free bread

**DOI:** 10.1038/s41598-025-10077-z

**Published:** 2025-07-08

**Authors:** Anna Pecyna, Monika Krzywicka, Agnieszka Buczaj, Agata Blicharz-Kania, Zbigniew Kobus

**Affiliations:** 1 Department of Technology Fundamentals, University of Life Sciences in Lublin, Głęboka 28, Lublin, 20-612 Poland; 2 Department of Biological Bases of Food and Feed Technologies, University of Life Sciences in Lublin, Głęboka 28, Lublin, 20-612 Poland

**Keywords:** Biotechnology, Chemical engineering

## Abstract

The aim of the study was to assess the effect of adding convectively dried and freeze dried raspberry and chokeberry pomace on the color (CIELab color space), texture (TPA test) and sensory evaluation (5-point scale) of gluten-free bread. Only in the case of bread with 10% addition of convectively dried chokeberry pomace, no statistically significant changes in color parameters were noted after baking and after 24 h. The highest increase in the lightness value (L*) (by 10.38%) was noted for gluten-free bread with 10% addition of freeze dried raspberry pomace, while the lowest (by 1.70%) was noted for bread with 5% addition of freeze dried raspberry pomace. In the case of enrichment of bread with chokeberry pomace, the highest increase in value (by 27.09%) was noted for bread with 10% addition of freeze dried chokeberry pomace, and the lowest (by 4.33%) for the control bread. The addition of raspberry pomace had no significant effect on the hardness and elasticity of the bread, but significantly reduced the cohesiveness of the crumb. The highest increase in hardness, by 95.9% compared to fresh bread, was observed for sample bread with 10% convection dried raspberry pomace addition (10CD_R). The introduction of raspberry pomace to the recipe did not significantly affect the chewiness of the bread, except for the sample with 10% addition of freeze dried pomace. In most cases, no significant increase in the chewiness of bread stored for 24 h was observed compared to fresh bread. In the case of chokeberry pomace, its addition did not cause significant changes in the elasticity and cohesiveness of the crumb, but a decrease in hardness and chewiness was noted. In the sensory assessment, the most tasty bread was considered to be the one with 10% addition of freeze dried raspberry pomace, while the least tasty bread was considered to be the one without raspberry and chokeberry pomace. The highest average of all parameter ratings was obtained by bread samples with a 5% addition of convection dried chokeberry pomace (5CD_C) (3.68).Gluten-free breads containing chokeberry and raspberry pomace could be implemented in bakery production, as they are accepted by consumers, but it is advisable to conduct further research on extending their shelf life.

## Introduction

 Agro-food production generates a very large amount of by-products and waste. Fruit residues can constitute up to 50% of the raw material. Fruit and food waste also arises as a result of damage during transport or storage. Plant by-products are rich in nutrients and active ingredients, such as proteins, unsaturated fatty acids, minerals, dietary fiber and antioxidant compounds. For this reason, fruit residues are used to design foods with functional properties, improving the nutritional value and health-promoting, technological and sensory properties of food products. Effective management of food waste as raw materials or food additives will have a beneficial effect on the health value of food, reduce nutritional problems and negative impacts on the environment and can be an economic benefit for producer. Efforts to achieve zero waste fit into the trend towards sustainable development and a circular economy and can have a direct impact on the Millennium Development Goals, the upcoming Sustainable Development Goals, the Post-2015 Agenda and the Zero Hunger Challenge^1–7^.

Poland is the world’s leading fruit producer, and the Lublin Province is one of the leading raspberry and chokeberry growing regions^8,9^. These fruits are mainly processed into juices, jams, teas, wines or liqueurs, during the production of which waste is produced in the form of pomace, peels and seeds, which are a good source of functional substances^10–13^. The pomace is characterised by a much higher content of valuable nutrients than the raw fruit and juice from which it was processed. Sójka et al.^14^ indicate that chokeberry pomace should be considered as a heterogeneous raw material. The seedless fractions are rich in fibre (~ 75%) and polyphenols, sodium, sorbitol and malic acid. The pomace containing seeds have a higher content of protein (24%), fat (13.9%), ash, K, Mg, Fe, Cu, Ca, citric acid, galacturonic acid, sucrose and glucose, but also have several times the content of amigdalin (120–180 mg/100 g dry weight), a cyanogenic glycoside^14^. These pomace are particularly useful in bakery products^15,16^ as they improve the sensory qualities and, due to their antioxidant content, also the shelf life of baked goods^17^. Raspberry pomace is an equally valuable source of bioactive components, but also a source of dietary fiber (59.5%) and high-quality seed oil (10%)^18^. According to Sójka et al.^19^ the average content of total polyphenols in seeds containing pomace was 3317 mg/100 g dry weight and in pomace without seeds 6895 mg/100 g dry weight. The pomace also contained 98% tannins (ellagotannins and proanthocyanidins).

Chokeberry and raspberry pomace can be an important addition to bakery products, and the product with high potential for fortification with plant material with high health-promoting value is gluten-free bread. Gluten-free breads are often of lower quality and nutritional value than breads made from conventional raw materials. Gluten plays a number of important roles in bread - from providing elasticity and structure to improving technological properties such as gas retention in the dough, which affects volume and texture. Bread texture is determined by examining features such as hardness (the force needed to compress the product with teeth), elasticity (the ability of the product to return to its original shape after deformation, e.g. biting), cohesiveness (the internal consistency of the product), chewiness (the amount of force needed to bite and prepare the product for swallowing - this is a combination of hardness, elasticity and cohesiveness)^20^. In turn, sensory acceptance of bread can be determined by analyzing properties such as: color (the color of the crust and crumb of the bread, which can affect the perceived freshness and nutritional value), aroma (the smell of the bread resulting from, among others, the ingredients used and fermentation processes), consistency (refers to the feel in the mouth and the structure of the bread, including softness, elasticity of the crumb), palatability (defined as the overall taste and smell impression when chewing and swallowing a bite of the product)^21,22^. These sensory attributes are experienced and evaluated through the human senses when the consumer interacts with the product and play an important role in the consumer’s overall perception of food. To increase nutritional value and improve textural and sensory properties, in addition to basic gluten-free ingredients such as gluten-free flour and starches, technologically and nutritionally functional ingredients are added to gluten-free bread^22^. One method of modifying gluten-free bread can be the use of fruit pomace, such as orange pomace^11,23^ apple pomace^24,25^ cherry pomace^26^ or raspberry pomace^27^. In the preparation of bakery products, fruit pomace, after being dried and ground into powder, is mixed with flour.

Although there are many drying methods, not every option allows for the dehydration of food while maintaining the required level of quality. The most commonly used drying technique is convection (hot air) drying. Unfortunately, long-term heating at high temperature, low dryer efficiency, and moderate process duration result in high energy consumption and a decrease in product quality^28^. Convection drying is often unable to meet the high quality requirements for the dried product because, despite a significant reduction in moisture content, their high temperature and prolonged exposure time often lead to the degradation of the relevant^29^. In turn, freeze-drying is considered one of the most effective methods of drying biological materials, especially when it comes to preserving nutritional values and sensory characteristics. However, from a technological point of view - due to high energy consumption and maintenance costs as well as extended processing time - it is not the most optimal solution, and is often a limiting factor for many producers^30,31^.

This article is a continuation of our previous studies on the evaluation of the effect of convectively dried and freeze dried raspberry pomace on the nutritional and antioxidant properties of gluten-free bread. In the article we proved the positive effect of adding raspberry residues on the amount of dietary fiber and active compounds in gluten-free bread^27^. From the consumer’s point of view, in addition to nutritional properties, the sensory properties of gluten-free bread are equally important, so it is important to check whether the addition of fruit pomace to bread will not cause deterioration of properties such as appearance, texture or palatability. Methods of preparing fruit pomace affect the chemical composition. However, the available literature reports have not compared the effects of raspberry and chokeberry pomace drying methods on the properties of gluten-free bread. Therefore, the purpose of this study is to evaluate the effect of the addition of convection dried and freeze dried raspberry and chokeberry pomace on the colour, texture and sensory evaluation of gluten-free bread.

## Materials and methods

### Raw materials

The research used raspberry fruits of the “*Polesie*” variety and chokeberry “*Aronia melanocarpa*”. The fruits came from an organic farm in the Lublin province. Immediately after harvesting, the raspberries and chokeberries were pressed on a basket press (810509 Browin, Łódź, Poland). Part of the obtained pomace was subjected to convectional drying using the Pol-Eko Aparatura SLW 115 Top + (Wodzisław Śląski, Poland; 48 h, 30 °C) and the second part was subjected to freeze-drying. Before freeze-drying, the raspberry pomace was frozen to a temperature of − 40 °C, as plates about 1 cm-thick, using a Memmert CTC256 climatic chamber (Schwabach, Germany). Then, the samples were freeze dried in a Martin ChristAlpha 2–4 LD plus device (Osterode am Harz, Germany) at a pressure of 20 Pa for 72 h. In order to preserve as many thermolabile bioactive compounds as possible during drying, the shelves were not heated. A laboratory grinder (Chemland, FW100, Stargard, Poland) was used to grind the pomace^27^. The pomace was sieved through a stainless steel sieve with a mesh size of 0.2 mm.

### Preparation of dough and bread baking procedure

The bread was prepared and marked according to the details given in Table 1. The dough was mixed in a laboratory spiral mixer (Kenwood, Havant, UK) for 5 min. The dough was then divided into 1075 g portions and placed in a mold for post-fermentation (40 min at 37 °C and 80% relative humidity). Afterward, the breads were baked in a convection-steam oven (Houno, Randers, Denmark) at 230 °C for 40 minutes^32^. After baking, the breads were left to cool naturally at room temperature for about 4 h. Some of the loaves were then used for testing, while the rest were packed in plastic bags. The appropriately marked samples were stored at room temperature, in a dry place.

**Table 1:** Model of experiment parameters.

Probe code	0_R/ 0_C	5CD_R/ 5CD_C	10CD_R/ 10CD_C	5FD_R/ 5FD_C	10FD_R/ 10FD_C
Ingredients	[%]				
Rice flour	50	45	40	45	40
Corn flour	40	40	40	40	40
Potato starch	10	10	10	10	10
Water	100	100	100	100	100
Rapeseed oil	6	6	6	6	6
Dry yeast	1.6	1.6	1.6	1.6	1.6
Salt	2.4	2.4	2.4	2.4	2.4
Sugar	2	2	2	2	2
Ground flax seeds	3	3	3	3	3
Convection dried raspberry/ chokeberry pomace	0	5	10	0	0
Freeze-dried raspberry/ chokeberry pomace	0	0	0	5	10

Percentages are calculated using the baker’s percentage method, where the weight of each ingredient is expressed as a percentage of the flour weight (the sum of all flours used), which is always 100%.

0_R – control bread; 5CD_R – bread with 5% convection dried raspberry pomace addition; 10CD_R – bread with 10% convection dried raspberry pomace addition.5FD_R – bread with 5% freeze dried raspberry pomace addition; 10FD_R – bread with 10% freeze dried raspberry pomace addition; 0_C – control bread; 5CD_C – bread with 5% convection dried chokeberry pomace addition; 10CD_C – bread with 10% convection dried chokeberry pomace addition 5FD_C – bread with 5% freeze dried chokeberry pomace addition; 10FD_C – bread with 10% freeze dried chokeberry pomace addition. Data values of each parameter with different superscript letters in rows are significantly different (Tukey test, *p ≤* 0.05).

### Color determination

The color parameters were tested 4 and 24 h after baking. To determine the color parameters, the bread was cut into slices about 2 cm thick. The color was measured on the surface without the crust, on the crumb, to avoid interference caused by possible browning. The color parameters were measured on each slice using a spectrophotometer in three places. Color was determined using a 3Color^®^ SF80 spectrophotometer. Each sample was analyzed in 10 replicates (light source: D65, observer: 10°, measuring head aperture diameter: 8 mm). The results were presented in the CIELab color space, where the following parameters were measured: L* – lightness, a* – color change from green to red and b* – color change from blue to yellow (higher values of a* and b* indicate more intense red and yellow). Additionally, color saturation C* was calculated using the formula: $\:{C}^{*}=\sqrt{{a}^{*2}+{b}^{*2}}$, and the total color change (absolute difference ∆*E*) between gluten-free bread (control sample) and individual bread samples with the addition of freeze dried and convection dried raspberry and chokeberry pomace was calculated, both after baking and after 24 h of storage, using the appropriate formula:

1 $$\:\varDelta\:E=\sqrt{{\left({\varDelta\:L}^{*}\right)}^{2}+{\left({\varDelta\:a}^{*}\right)}^{2}+{\left({\varDelta\:b}^{*}\right)}^{2}}$$

where ∆*L*, ∆*a*, and ∆*b* — indices of the difference in the color of the surfaces of samples compared with the control bread^32^.

### Determination of textural properties of bread

Four hours after baking, the texture of the bread crumb was evaluated using a TPA test. A testing machine (Zwick/Roel, Z0.5, AG, Ulm, Germany) with TestXpert II software was used for the analysis. To prepare samples for textural tests, loaves of bread were cut into 10 mm thick slices immediately before the test. The outermost slices were discarded. Then, cuboids measuring 30 × 30 × 10 mm were cut from the middle part of the slices. During the test, the product was compressed twice to 50% of its original height, using a 50 kg load cell. The compression speed was set at 50 mm·min^*−*1^. A flat cylindrical punch with a 100 mm diameter was used for the compression. The test measured the following parameters: hardness [N], elasticity [-], cohesiveness [-], and chewiness [N]^33^. Each sample was analyzed in 10 replicates. The same tests were performed 24 h after baking.

### Determination of sensory parameters of bread

The study was approved by the University Ethics Committee for Human Research at the University of Life Sciences in Lublin (resolution number UKE/32/2024). All methods were carried out in accordance with relevant guidelines and regulations. Breads with added chokeberry and raspberry pomace were assessed by a panel of 52 trained consumers selected from among employees and students of the University of Life Sciences in Lublin (aged 21–64). The selection criteria were: good health, no smoking and voluntary participation. Consumers expressed interest in consuming gluten-free bread enriched with additives that potentially improve its structural and flavor properties. We obtained informed consent from all participants to participate in the study. The study participants were trained in the sensory evaluation methodology by the study authors. They were introduced to the sensory evaluation procedure, the meaning of individual attributes used in the descriptive analysis, the method of tasting individual samples, and the method of recording the evaluation results in accordance with the adopted coding method. The study was conducted in a laboratory under LED lighting and at room temperature. Participants were given mineral water as a neutralizing agent. Samples were given in random order. The assessment was carried out immediately after baking and cooling the bread. Immediately prior to the sensory evaluation, bread crumb samples measuring 2 × 2 × 2 cm were prepared, placed on paper trays and coded accordingly. Participants assessed the color, smell, consistency and palatability of the pulp. The results were presented on a 5-point structural scale (1 – “I really don’t like it” and 5 – “I really like it”).

### Statistical analyses

The obtained results were subjected to analysis of variance (ANOVA). The significance of the variations among the mean values was assessed using Tukey’s test at a significance level of *p* < 0.05. Additionally, the principal component analysis (PCA) was carried out. All statistical analysis were performed with Statistica software (Statistica 13; StatSoft Inc., Tulsa, OK, USA).

## Results

### The color of bread

**Fig. 1:**
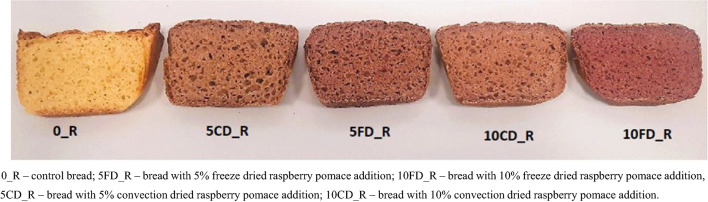
Control and gluten-free breads fortified with raspberry pomace.

The results regarding the color of gluten-free breads with the addition of raspberry pomace are presented in Fig. 1 and provided in Table 2.

Table 2 presents the results of the color analysis of gluten-free bread with the addition of convection dried and freeze dried raspberry pomace and the control sample. The use of raspberry pomace additives caused significant changes in color parameters (L*, a*, b*, C*). The highest lightness value (L*) measured after baking was recorded for the control bread. Breads fortified with raspberry pomace were characterized by a statistically significantly lower value of the L* parameter. In the case of bread with the addition of 10% convection dried raspberry pomace (10CD_R), a decrease in the value was noted after 24 h, while in the remaining cases, there was an increase in lightness after 24 h. The color of the bread became darker with the increase in the level of pomace addition. However, significant changes were observed only for samples 0_R and 10 FD_R. The highest increase in value was noted for gluten-free bread with 10% freeze dried raspberry pomace (10FD_R) by 10.38%, and the lowest for bread with 5% freeze dried raspberry pomace (5FD_R) by 1.70%. Based on the analysis, it can be concluded that there are no statistically significant differences between the lightness of bread (parameter L*) measured immediately after baking and 24 h after baking for 5FD_R, 5CD_R, 10CD_R bread. The lightness of bread with pomace added measured immediately after baking is statistically significantly different for 10FD_R bread and measured 24 h after baking for 10CD_R bread.

**Table 2:** Color parameters obtained from the crumb of gluten-free bread with the addition of raspberry pomace measured after 4 and 24 h after baking.

Probe	Time [h]	L* - value	a* - value	b* - value	C* - value	∆E - value
0_R	4	58.70 ± 2.01^b^	1.89 ± 0.11^g^	26.07 ± 0.45^a^	26.14 ± 0.45^a^	0
5CD_R	4	46.53 ± 1.45^def^	7.55 ± 0.19^e^	16.98 ± 0.10^c^	18.58 ± 0.14^d^	19.93
10CD_R	4	45.77 ± 0.74^ef^	9.91 ± 0.35^c^	16.49 ± 0.35^c^	19.24 ± 0.39^c^	21.59
5FD_R	4	47.49 ± 0.86^cde^	7.65 ± 0.19^e^	15.42 ± 0.39^d^	17.21 ± 0.39^e^	19.84
10FD_R	4	43.82 ± 1.15^g^	11.58 ± 0.34^a^	12.49 ± 0.37^g^	17.03 ± 0.46^ef^	25.58
0_R	24	63.78 ± 0.92^a^	1.61 ± 0.23^g^	25.01 ± 0.66^b^	25.06 ± 0.66^b^	0
5CD_R	24	47.40 ± 1.30^cde^	6.20 ± 0.27^f^	14.85 ± 0.18^d^	16.09 ± 0.21^g^	19.81
10CD_R	24	45.22 ± 1.07^fg^	8.67 ± 0.28^d^	13.95 ± 0.18^f^	16.42 ± 0.21^fg^	22.73
5FD_R	24	48.28 ± 1.00^cd^	7.37 ± 0.56^e^	14.53 ± 0.67^f^	16.30 ± 0.77^g^	19.57
10FD_R	24	48.37 ± 1.08^c^	10.78 ± 0.23^b^	11.76 ± 0.27^h^	15.96 ± 0.31^g^	22.30

0_R – control bread; 5FD_R – bread with 5% freeze dried raspberry pomace addition; 10FD_R – bread with 10% freeze dried raspberry pomace addition, 5CD_R – bread with 5% convection dried raspberry pomace addition; 10CD_R – bread with 10% convection dried raspberry pomace addition. Data values of each parameter with different superscript letters in rows are significantly different (Tukey test, *p ≤* 0.05).

The differences in the lightness of bread with the addition of convection dried (CDR) and freeze dried (FDR) raspberry pomace can be explained by the specificity of the drying processes and their effect on the stability of pigment compounds, mainly anthocyanins. It can be assumed that convective drying, which takes place at an elevated temperature, leads to significant degradation of these heat-sensitive pigments. As a result, the colour of the pomace becomes lighter, which translates into a higher lightness (L*) of the bread with its addition - especially noticeable at a higher, 10% level of pomace.

On the other hand, freeze-drying, thanks to the use of low temperatures and vacuum, allows for much better preservation of anthocyanins and other bioactive compounds. FDR pomace retains a more intense colour, which results in a lower lightness of the bread, especially with a higher addition (10%), where the difference becomes more visible. The behavior of bread brightness at different levels of pomace addition may also be related to the non-linear effect of dyes as a function of their concentration – at lower additions (5%) the differences may be masked by the remaining dough ingredients, while at 10% the influence of the pomace color properties becomes more dominant.

The lowest value of the a* parameter indicating the intensity of the red color was recorded for the control bread. The addition of raspberry pomace caused an increase in the a* parameter value. The highest value was recorded for the bread with the addition of 10% freeze dried raspberry pomace (10FD_R). In all cases, a decrease in the intensity of the red color was noted after 24 h of storage. The highest decrease was noted for the bread with the addition of 5% convection dried raspberry pomace (5CD_R) (by 17.88%), and the lowest for the bread with the addition of 5% freeze dried raspberry pomace (5FD_R) (by 3.66%). In the case of the a* parameter measured immediately after baking and after 24 h of storage, no statistically significant differences were noted for the control bread and the bread with the addition of 5% freeze dried raspberry pomace (5FD_R). Regardless of the drying method of the by-products, the a* parameter values measured after baking do not differ statistically significantly for the bread with the addition of 5% raspberry pomace.

The highest value of the b* parameter indicating the intensity of the yellow color measured after baking was recorded for the control bread. Bread fortified with raspberry pomace were characterized by a statistically significantly lower intensity of the yellow color. In all cases, a decrease in the intensity of the yellow color was noted after 24 h. The highest decrease was noted for bread with the addition of 5% convection dried raspberry pomace (5CD_R) (by 12.54%), and the lowest for the control bread (by 4.06%). The b* parameter for all the breads tested differed statistically significantly after baking and 24 h after baking. In the case of the b* parameter measured immediately after baking, no statistically significant differences were noted for bread with the addition of 5% and 10% convection dried pomace. The b* parameter values measured after 24 h do not differ significantly between bread with the addition of 5% pomace.

Bread fortified with raspberry pomace was characterized by a statistically significantly lower value of the C* parameter reflecting the color saturation than the control bread. In all cases, a statistically significant decrease in the C* parameter value was noted after 24 h. The highest C* parameter value was noted for the control bread. The lowest C* parameter value among bread enriched with raspberry pomace was noted for bread with the addition of 10% freeze dried raspberry pomace. The absolute color difference criterion (∆*E*) was also used to develop the results, which indicates the differences between the colors of two samples (the higher the value, the greater the difference). The color of the crumb of the bread fortified with pomace was compared with the color of the control bread. In all samples of bread with the addition of raspberry pomace, the ∆*E* value obtained is greater than 5, which indicates a significant color deviation (the observer has the impression of two different colors). The greatest difference in the color of fresh bread was noted for sample 10 FD_R. On the other hand, the greatest decrease in the color difference parameter value after baking and after 24 h of storage was noted for bread fortified with 5% addition of freeze dried raspberry pomace (5FD_R) by 12.8%, while the lowest decrease in the ∆*E* value after baking and after 24 h was obtained for gluten-free bread with 5% addition of convection dried raspberry pomace (5CD_R) – a decrease of 0.6%. Only in the case of bread with a 10% addition of convection dried raspberry pomace (10CD_R) there was an increase in the absolute color difference criterion by 5.28%.

**Fig. 2:**
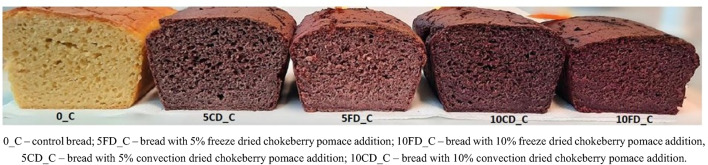
Control and gluten-free breads fortified with chokeberry pomace.

The results regarding the color of gluten-free breads with the addition of chokeberry pomace are presented in Fig. 2 and provided in Table 3.

**Table 3:** Color parameters obtained from the crumb of gluten-free bread with the addition of chokeberry pomace measured after 4 and 24 h after baking.

Probe	Time [h]	L* - value	a* - value	b* - value	C* - value	∆E - value
0_C	4	60.94 ± 3.09^a^	1.83 ± 0.33^f^	25.84 ± 0.87^a^	25.90 ± 0.86^a^	
5 CD_C	4	32.07 ± 2.05^d^	9.05 ± 0.45^d^	5.99 ± 0.81^d^	10.86 ± 0.75^f^	37.29
10 CD_C	4	26.06 ± 2.65^fg^	10.03 ± 0.28^c^	2.50 ± 0.35^f^	10.34 ± 0.27^f^	44.29
5 FD_C	4	36.03 ± 2.50^c^	10.25 ± 0.29^c^	8.03 ± 0.26^c^	13.02 ± 0.32^d^	33.25
10 FD_C	4	24.77 ± 1.89^g^	12.72 ± 0.98^b^	3.21 ± 0.47^ef^	13.12 ± 0.95^d^	45.65
0_C	24	63.58 ± 1.03^a^	1.31 ± 0.18^f^	24.37 ± 0.73^b^	24.41 ± 0.73^b^	
5 CD_C	24	38.13 ± 1.97^bc^	7.35 ± 0.38^e^	5.92 ± 0.46^d^	9.44 ± 0.53^g^	32.01
10 CD_C	24	28.93 ± 1.61^ef^	9.64 ± 0.58^cd^	2.65 ± 0.46^f^	10.01 ± 0.62^fg^	41.73
5 FD_C	24	40.67 ± 2.18^b^	9.17 ± 0.34^d^	7.86 ± 0.42^c^	12.09 ± 0.29^e^	29.31
10 FD_C	24	31.48 ± 1.12^de^	13.55 ± 0.31^a^	3.63 ± 0.20^e^	14.03 ± 0.29^c^	40.13

0_C – control bread; 5FD_C – bread with 5% freeze dried chokeberry pomace addition; 10FD_C – bread with 10% freeze dried chokeberry pomace addition, 5CD_C – bread with 5% convection dried chokeberry pomace addition; 10CD_C – bread with 10% convection dried chokeberry pomace addition. Data values of each parameter with different superscript letters in rows are significantly different (Tukey test, *p ≤* 0.05).

Table 4 presents the results of the color analysis of gluten-free bread with the addition of both convectively dried and freeze dried chokeberry pomace and the control sample. The highest value of the L* parameter, i.e. color brightness, measured after baking was recorded for the control bread. Bread fortified with chokeberry pomace was characterized by statistically significantly lower brightness. In all cases, an increase in brightness (L* parameter value) measured after 24 h of storage was recorded. The highest increase in value was recorded for bread with the addition of 10% freeze dried chokeberry pomace (10FD_C) by 27.09%, and the lowest for the control bread (0_C) by 4.33%. The analysis did not reveal any statistically significant differences in the L* parameter measured immediately after baking and after 24 h, both in the bread enriched with 10% convection dried chokeberry pomace (10CD_C) and in the control sample (0_C). The lightness of the crumb of bread with the addition of 10% chokeberry pomace (dried using different methods) did not differ significantly for both fresh bread and bread stored for 24 h.

**Table 4:** Textural properties of gluten-free breads with raspberry pomace addition measured after 4 and 24 h after baking.

Probe	Time [h]	Hardness [*N*]	Elasticity [-]	Cohesiveness [-]	Chewiness [*N*]
0_R	4	15.96 ± 1.50^cd^	0.84 ± 0.02^abcd^	0.40 ± 0.01^c^	5.18 ± 0.47^c^
5CD_R	4	14.00 ± 0.58^cd^	0.86 ± 0.04^ab^	0.43 ± 0.02^bc^	5.13 ± 0.58^c^
10CD_R	4	13.56 ± 1.11^d^	0.79 ± 0.03^cde^	0.47 ± 0.01^a^	5.08 ± 0.46^c^
5FD_R	4	14.14 ± 0.84^cd^	0.84 ± 0.01^abcd^	0.43 ± 0.01^abc^	5.09 ± 0.38^c^
10FD_R	4	17.04 ± 0.27^c^	0.87 ± 0.03^a^	0.45 ± 0.02^ab^	6.72 ± 0.43^ab^
0_R	24	28.64 ± 1.51^a^	0.77 ± 0.01^e^	0.23 ± 0.01^g^	5.16 ± 0.11^c^
5CD_R	24	24.38 ± 1.03^b^	0.82 ± 0.03^abcde^	0.29 ± 0.03^f^	5.86 ± 0.73^bc^
10CD_R	24	26.56 ± 3.21^ab^	0.78 ± 0.02^de^	0.37 ± 0.04^de^	7.81 ± 0.69^a^
5FD_R	24	24.50 ± 1.57^b^	0.80 ± 0.04^bcde^	0.31 ± 0.03^f^	6.10 ± 0.76^bc^
10FD_R	24	25.76 ± 1.37^ab^	0.84 ± 0.03^abc^	0.34 ± 0.01^ef^	7.29 ± 0.23^a^

0_R – control bread; 5FD_R – bread with 5% freeze dried raspberry pomace addition; 10FD_R – bread with 10% freeze dried raspberry pomace addition, 5CD_R – bread with 5% convection dried raspberry pomace addition; 10CD_R – bread with 10% convection dried raspberry pomace addition. Data values of each parameter with different superscript letters in rows are significantly different (Tukey test, *p ≤* 0.05).

The lowest value of the a* parameter was recorded for the control bread. The addition of chokeberry pomace caused an increase in the a* parameter value. The highest value was recorded for bread with the addition of 10% freeze dried chokeberry pomace (10FD_C). In the case of 10FD_C bread, an increase in the a* parameter value was noted after 24 h, while in the remaining cases, a decrease was noted. The largest decrease in value was observed in the control bread and amounted to 28.42%, and the smallest in the bread with 10% convection dried chokeberry pomace (10CD_C) – 3.89%, and this was a statistically insignificant change. Based on the analysis, it can be concluded that there are no statistically significant differences between the a* parameter measured after baking and 24 h after baking for the control bread and the 10CD_C bread. The results of the a* parameter measurements conducted immediately after baking and after 24 h did not show statistically significant differences between the bread with 5% freeze dried chokeberry pomace (5FD_C) and the bread with 10% convection dried chokeberry pomace (10CD_C).

The highest value of the b* parameter measured after baking was recorded for the control bread. Bread fortified with chokeberry pomace were characterized by a statistically significantly lower value of the b* parameter. The higher the share of pomace, the lower the values of the b* parameter. The b* parameter after baking and 24 h of storage did not differ statistically significantly for all breads with the addition of chokeberry pomace. In the case of the b* parameter measured immediately after baking, no statistically significant differences were noted for bread with the addition of 10% pomace.

The highest value of the C* parameter referring to color saturation was recorded for the control bread. Bread fortified with chokeberry pomace was characterized by a statistically significantly lower value of the C* parameter than the control bread. Bread fortified with convectively dried pomace was characterized by a lower C* parameter compared to the product containing freeze dried pomace. Increasing the share of pomace from 5 to 10% did not cause significant changes in the tested parameter. In the case of bread with the addition of 10% convectively dried chokeberries, no statistically significant differences were noted after 24 h. There were no statistically significant differences between bread with the addition of 5% and 10% convection dried chokeberries after 24 h after baking.

As for the parameter concerning the absolute color difference (∆*E*), during the tests of gluten-free bread with the addition of chokeberry pomace, similar results were obtained as in the tests of bread with the addition of raspberry pomace, i.e. the value of the ∆*E* parameter is greater than 5, which indicates a significant deviation in color (the observer has the impression of two different colors). These changes apply to all analyzed samples. In all samples, a decrease in the color difference value was noted after 24-hour storage. The highest decrease in the color difference parameter value after baking and after 24 h of storage was noted for bread fortified with 5% convection dried raspberry pomace (5CD_R) by 14.2%, while the lowest decrease in the ∆*E* value after baking and after 24 h was obtained for gluten-free bread with 10% convection dried raspberry pomace (10CD_R) – a decrease of 5.8%.

Raspberry pomace, like other sources of natural additives, such as chokeberry or elderberry, has gained recognition in the food industry due to its color stability and its relatively low price and high availability^34,35^. It offers a variety of coloring and health properties that make it attractive to food producers. There are few studies available in the literature on the addition of fruit pomace to gluten-free bread. In the studies on wafers fortified with freeze dried raspberry pomace, conducted by Szymanowska et al.^10^similarly to the a* parameter) as in our work, it was shown that with the increase in the share of pomace in the wafer cookie, the color became darker (decrease in the L* parameter) and redder (increase)^10^. In turn, Šarić et al.^36^ used convection dried raspberry and blueberry pomace to enrich gluten-free cookies. Their studies indicate that replacing 30% of the gluten-free flour mixture with a different proportion of raspberry and blueberry pomace in gluten-free cookies also led to increased darkness and redness of the cookies and reduced yellowness^10,36^. Similarly, in our studies, the addition of raspberry pomace increased the redness of gluten-free bread. This is related to the presence of the characteristic red pigment cyanoside-3-sophoroside^37^. After six months of storage, Šarić et al.^36^ observed a change in the color parameter ∆*E* in the range of 6.0–12.0 in gluten-free cookies with the addition of raspberry pomace of 22.5% and 30%. On the other hand, cookies with the addition of blueberry pomace also showed noticeable color changes during storage, but the average ∆*E* value was 1.57, which indicates a higher stability of anthocyanins from blueberries compared to those from raspberry pomace.

In the case of chokeberry pomace, we assume that the dyes behaved more stably than the pigments contained in raspberry pomace. We therefore conclude that the longer time and higher temperature of convective drying caused the chokeberry pomace to darken. We suspect that apart from to pigment degradation, Maillard reactions and the caramelization process may also be responsible for the color changes of chokeberries after drying processes. During the freeze-drying process, chokeberry pomace is not subjected to such a high temperature, hence the dyes are not degraded.

In the study by Różyło et al.^38^ on the effect of adding freeze dried chokeberry and elderberry powder to gluten-free wafers, it was found that the addition of elderberry caused significant darkening, compared to chokeberry, in the range of 3 to 5%. However, a greater redness (a*) of the wafers was obtained with 1–5% chokeberry content. Elderberry powder contains larger amounts of blue pigments, which affect the color of the wafers after baking, giving them a characteristic shade. Hence, compared to chokeberry, significantly lower values of the b* parameters were obtained for wafers with 2, 3, 4, and 5% addition of elderberry powder. The color parameter ∆*E* was significantly higher for freeze dried elderberry powder. It can be assumed that the pigments present in elderberry fruits are characterized by greater stability than those found in chokeberries^38^. Similar relationships were observed during studies on wheat bread. Guijarro-fuertes et al.^39^ added Andean blueberries to wheat bread, which reduced the lightness (L*), redness (a*), and yellowness (b*) of the bread crust. Hashemi et al.^40^ indicated that the inclusion of goji berry powder in wheat bread also reduced L* and a*. However, the reduction in L* can be attributed to the accelerated Maillard reaction caused by increased substrate availability and the reddish color of goji berry powder. Composite bread containing goji berry powder has a darker color compared to the control wheat bread, which is attributed to beta-carotene pigments in the fruit^40^. Although fruit concentrates containing anthocyanins can be effectively used as natural food colors, their use is limited by low stability and color changes that may affect the perceived quality of the final product by consumers. In turn, Sadowska et al.^41^ observed in their study that the brownish color of chokeberry pomace powder was the result of processes such as water evaporation, dye concentration, enzymatic browning and oxidation reactions occurring during drying. Attention should also be paid to significant color changes during storage, which may be related to the degradation of polyphenols in stored bakery products. The color and stability of antioxidants, especially the least stable anthocyanins, are influenced by several factors, such as pH, storage and processing temperature, chemical structure, concentration, UV light, oxygen, solvents, the presence of enzymes, copigments, proteins and metal ions^38^.

### Textural properties of bread

Table 4 shows the crumb texture of gluten-free bread prepared by replacing rice flour with convection dried and freeze dried raspberry pomace in the amounts of 5 and 10%.

One of the basic mechanical parameters of bakery products is hardness, defined as the force required for deformation into the product. In other words, it is the amount of force required for the first deformation of a piece of bread during chewing^32^. It was observed that the addition of convectively dried fruit residues caused a slight decrease in the hardness of the crumb, from 15.96 N to 13.56 N. However, statistical analysis did not confirm the significance of these changes. It should therefore be stated that the hardness of fresh gluten-free bread fortified with raspberry pomace did not change significantly. However, during storage the hardness of the bread crumb changed significantly compared to fresh bread (regardless of the amount and method of drying the raspberry pomace). It is worth noting that the highest hardness was noted for sample 0_R, while the greatest increase in hardness, by 95.9% compared to fresh bread, was observed for sample 10CD_R. Crumb hardening is caused by staling, which is a complex phenomenon with many mechanisms at work. Factors influencing bread crumb staling include starch retrogradation. Water also plays a key role in bread staling due to its plasticizing effect on the crumb network. Water loss from the crumb during storage, via diffusion from the crumb to the crust, probably also contributed to crumb hardening^42^.

Elasticity means the ability to regain shape after deformation. The changes in the elasticity of the crumb of gluten-free bread were not large, for fresh bread they were at the level of 0.79–0.87 for samples 10CD_R, 10FD_R, respectively. It can therefore be concluded that increasing the addition of convection dried raspberry pomace (from 5 to 10%) had a negative effect on the elasticity of gluten-free bread. On the other hand, introducing freeze dried pomace into the recipe did not cause any significant changes in this parameter. After storing the bread for 24 h, its elasticity generally decreased. However, significant changes were observed only for sample 0_R. The parameter value decreased by an average of 8.3%.

The cohesiveness value (from 0 to 1) describes the degree to which a product piece holds together as a whole. It is related to the degree to which a material can be deformed but not broken. Cohesion increased after introducing the addition of dried pomace. However, these changes were significant only after increasing the share of the freeze dried by-product to 10%. The crumb of gluten-free bread stored for 24 h was characterized by lower cohesion than that of fresh bread. A significant decrease in the parameter value was observed regardless of the amount and method of drying raspberry pomace. The highest cohesion values after 24 h were recorded for sample 10CD_R.

Chewiness is an important textural parameter. It is understood as the value of the force needed to chew one bite of food before swallowing. Chewiness depends on other parameters, such as hardness, elasticity, or cohesiveness. Too high a value of this parameter may indicate difficulties in consuming food. Introducing raspberry pomace to the gluten-free bread recipe generally did not significantly affect the changes in chewiness. The exception is the test with the addition of 10% freeze dried fruit residues, then an increase in the tested parameter was observed. It should also be noted that for most of the tests, no significant increase in chewiness was observed in bread stored for 24 h, compared to fresh bread. Only the crumb of the 10CD_R bread was characterized by greater chewiness after storage.

According to the analysis of the conducted studies, it should be stated that the addition of raspberry pomace to the gluten-free bread recipe does not significantly affect the changes in hardness, elasticity or chewiness of the crumb. Changes in the last two parameters were considered significant only for one of the tests, where the share of by-products was 10%. The presented observations are similar to the results of Mildner-Szkudlarz et al.^43^. In the authors’ experiment, the texture of muffins with the addition of 5 and 10% of convection dried raspberry pomace was examined. The muffins were baked in different conditions. It was found that hardness, chewiness, and cohesiveness did not change significantly regardless of the amount of raspberry pomace for a given baking method. In turn, in our experiment, it was shown that the addition of 10% of raspberry residue caused an increase in cohesiveness. Changes in this parameter may be positively perceived by consumers. Cohesiveness is related to the strength of internal bonds between ingredients, therefore its increase will have a positive effect on the maintenance of the product as a whole^44^. It was also observed that at 10% raspberry pomace level, the use of convection dried pomace did not change the chewiness of the product compared to the control; however, the use of freeze dried pomace increased the chewiness of the bread compared to the control. Freeze dried pomace is characterized by lower moisture and contains more dietary fiber^27^. Therefore, it has a higher capacity to absorb water from the dough, which probably worsens the gelatinization of starch and results in an increase in the hardness of the bread crumb^45^. Chewiness is the amount of force needed to prepare a food bolus for swallowing and is closely related to the hardness of the material. Hence, increasing the hardness of the 10FD_C sample (although statistically insignificant) influenced significant changes in the chewiness of the sample.

Another important feature is elasticity, or the ability of the material to return to its original shape after stress. As mentioned, its changes were not significant in the described study. Changes in this parameter may vary depending on the type of pomace. In the studies of Rocha Parra et al.^44^ a larger amount of apple pomace results in lower elasticity. Similar changes in elasticity caused by the addition of apple pomace (min. 15%) are observed by Kırbas¸ et al.^46^. The authors also proved that even a 5% share of carrot and orange pomace results in a decrease in the elasticity of gluten-free dough.

Table 5 shows the crumb texture of gluten-free bread prepared by replacing rice flour with convection dried and freeze dried chokeberry pomace in the amount of 5 and 10%.

**Table 5:** Textural properties of gluten-free bread with chokeberry pomace addition measured after 4 and 24 h after baking.

Probe	Time [h]	Hardness [*N*]	Elasticity [-]	Cohesiveness [-]	Chewiness [*N*]
0_C	4	13.54 ± 0.36^e^	0.85 ± 0.03^a^	0.43 ± 0.03^ab^	4.71 ± 0.38^bc^
5CD_C	4	8.77 ± 0.31^f^	0.85 ± 0.01^a^	0.44 ± 0.02^a^	3.24 ± 0.15^e^
10CD_C	4	11.21 ± 0.77^a^	0.81 ± 0.02^ab^	0.41 ± 0.02^ab^	3.70 ± 0.26^de^
5FD_C	4	9.34 ± 1.44^a^	0.82 ± 0.04^ab^	0.39 ± 0.03^b^	2.96 ± 0.58^e^
10FD_C	4	15.70 ± 0.63^de^	0.78 ± 0.04^ab^	0.41 ± 0.01^ab^	5.06 ± 0.22^abc^
0_C	24	29.52 ± 1.44^a^	0.78 ± 0.03^ab^	0.23 ± 0.01^d^	5.40 ± 0.38^ab^
5CD_C	24	18.76 ± 1.18^c^	0.74 ± 0.07^b^	0.27 ± 0.02^cd^	3.69 ± 0.50^de^
10CD_C	24	19.52 ± 0.77^c^	0.81 ± 0.04^ab^	0.28 ± 0.02^c^	4.41 ± 0.34^cd^
5FD_C	24	17.94 ± 0.63^cd^	0.80 ± 0.04^ab^	0.24 ± 0.02^cd^	3.39 ± 0.33^e^
10FD_C	24	26.14 ± 0.68^b^	0.83 ± 0.06^ab^	0.27 ± 0.02^cd^	5.74 ± 0.30^a^

0_C – control bread; 5FD_C – bread with 5% freeze dried chokeberry pomace addition; 10FD_C – bread with 10% freeze dried chokeberry pomace addition, 5CD_C – bread with 5% convection dried chokeberry pomace addition; 10CD_C – bread with 10% convection dried chokeberry pomace addition. Data values of each parameter with different superscript letters in rows are significantly different (Tukey test, *p ≤* 0.05).

Gluten-free breads containing added chokeberry pomace were characterized by lower hardness compared to the control bread. These changes were significant for most samples, except for 10FD_C. Similar dependencies were observed when assessing the hardness of bread stored for 24 h. The highest crumb hardness was noted for the control bread − 29.52 N.

The elasticity of the crumb of gluten-free bread did not change significantly after introducing chokeberry pomace to the recipe. The highest values of this parameter were noted for fresh bread samples 0_C and 10CD_C. After storage, a decrease in the elasticity of the crumb was observed. However, statistical analysis showed significant changes, compared to fresh bread, only for sample 5CD_C.

Regardless of the amount and method of drying chokeberry pomace, its addition to gluten-free bread did not significantly affect the changes in its cohesiveness. A significant decrease in the parameter occurred after 24 h of bread storage, regardless of the probe. The cohesiveness of the bread after 24 h was similar for all probes. The significantly highest values were recorded for bread with the addition of 10% convection dried chokeberry pomace.

The introduction of chokeberry pomace to the gluten-free bread recipe generally resulted in a significant decrease in chewiness. Only for the 10FD_C test were no significant changes in the parameter observed. It should be noted that the bread stored for 24 h was characterized by similar chewiness to fresh bread. The changes in the parameters were not significant within the individual recipes. The highest chewiness was recorded for the 0_C and 10FD_C tests.

Fortification of gluten-free bread with chokeberry pomace did not cause significant changes in crumb elasticity and cohesiveness. However, a decrease in the level of hardness and chewiness was observed. In previous studies, the use of chokeberry pomace in bakery products usually increased the hardness of the product. Such a relationship was noted by Cacak-Pietrzak et al.^47^ and Zbikowska et al.^48^. However, it should be noted that the subject of the authors’ research was wheat bread and muffins. In another experiment, in which the properties of gluten-free bread with the addition of fruit pomace were examined, a decrease in crumb hardness was observed after the introduction of a small amount of the by-product O’shea et al.^23^. Orange pomace reduced the hardness of the gluten-free bread crumb texture to a certain point, but further increases in levels led to a significant increase in the hardness value. In turn, in the study by Šarić et al.^36^ replacing the gluten-free flour mixture with raspberry pomace resulted in lower cookie hardness values than those obtained with blueberry pomace. These changes were probably due to the different water retention during baking of the different fruit by-products. In general, gluten-free breads are characterized by a higher crumb hardness and an increased rate of hardening compared to wheat breads (which justifies double tests of textural properties - after a few and then 24 h after baking). The reason for the high intensity of these changes is primarily the lack of gluten in the starch matrix, which, as has been shown, delays staling due to interactions it has with starch^23^. Since most gluten-free breads contain a high percentage of starch and a low percentage of protein, staling occurs faster. It should be noted that the addition of dried fruit or their residues slows down the staling processes and allows for maintaining a lower crumb hardness. This relationship can be observed by comparing samples 0_R and 10FD_R and 0_C and 10FD_C. Analogous changes were observed in the experiment by Gumul et al.^26^. According to the authors, the addition of cherry pomace (10 and 20%) to gluten-free bread caused a decrease in the hardness of the bread stored for 2 and 3 days.

### Sensory analysis of bread

**Fig. 3:**
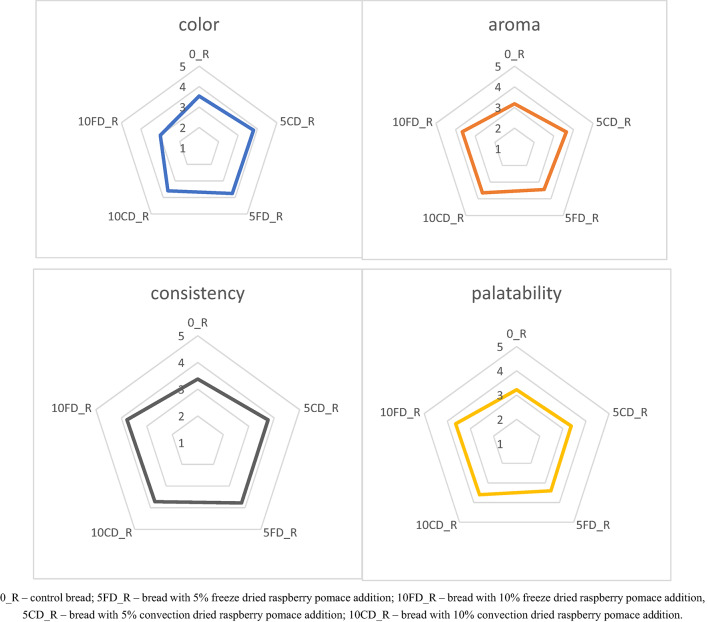
Sensory evaluation of gluten-free bread with added raspberry pomace.

The results of the sensory evaluation of gluten-free breads with raspberry pomace are presented in Fig. 3. Consumers indicated that the most delicious bread was the one with 10% freeze dried raspberry pomace (10FD_R) and the one with 10% dried raspberry pomace (10CD_R). According to the assessors, the best consistency was that of the bread with 10% and 5% freeze dried raspberry pomace (10FD_R, 5FD_R) and with 5% dried raspberry pomace (5CD_R). The smell was rated the highest for the sample with 10% freeze dried raspberry pomace (10FD_R). The worst taste, consistency and smell, according to consumers, was that of the control bread (0_R). The most beautiful color was characteristic of the bread with 5% addition of dried raspberry pomace (5CD_R), and the worst score in this respect was obtained by the bread with 10% freeze dried raspberry pomace (10FD_R). The analysis of variance showed that there is a statistically significant difference in the average color assessment. In the Tukey analysis, 2 homogeneous groups were distinguished. The average color assessments for the control bread and 10FD_R do not differ significantly. There are also no statistically significant differences between the control bread, 5CD_R, 10CD_R and 5FD_R. For the variables aroma, consistency, and palatability, no statistically significant differences were noted between the breads.

**Fig. 4:**
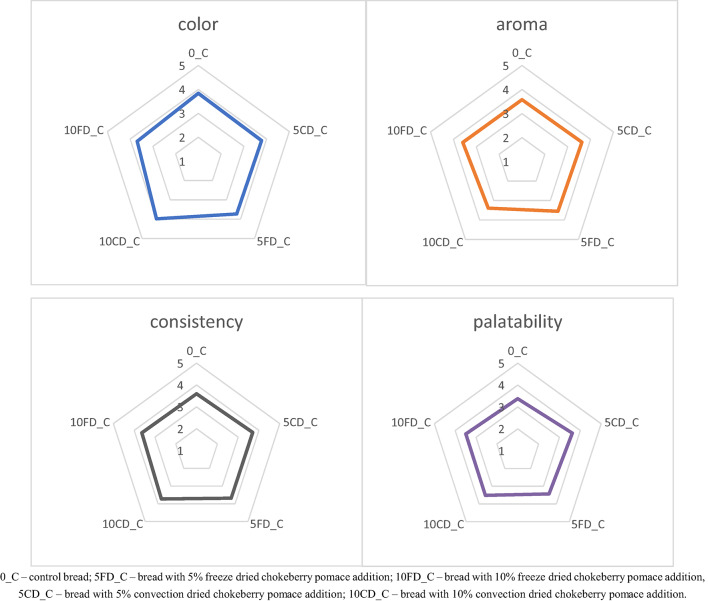
Sensory evaluation of gluten-free bread with added chokeberry pomace.

The results of the sensory evaluation of gluten-free breads with chokeberry pomace are presented in Fig. 4. Consumers indicated that the bread with 5% convection dried chokeberry pomace (5CD_C) was the tastiest and most aromatic. Consumers indicated that the bread without additives (0_C) was the least tasty, and this bread was also assessed as the worst in terms of consistency. Consumers assessed that the bread with 10% convection dried chokeberry pomace (10CD_C) was the least aromatic, but had the best color and consistency. The bread with 10% freeze dried chokeberry pomace (10FD_C) had the worst color in the consumers’ assessment. The analysis of variance showed that there are no statistically significant differences for the variables color, smell, consistency and palatability between the breads.

When comparing breads with added chokeberry and raspberry pomace, they were rated similarly in terms of taste. Bread with added raspberry pomace was considered more aromatic and with better consistency, and bread with chokeberry pomace had better color.

Of all the samples, the tastiest were indicated as bread with a 10% addition of freeze dried raspberry pomace (10FD_R) (score 3.64), with a 5% addition of dried chokeberry pomace 5CD_C (3.62) and 5% addition of freeze dried raspberry pomace 5FD_R (3.60). The worst in terms of taste were rated bread without added chokeberries (3.38) and raspberries (3.22). The highest average of all parameter ratings was obtained by bread samples with a 5% addition of convection dried chokeberry pomace 5CD_C (3.68). This bread also received the highest total of partial scores (14.72). According to Difonzo et al.^49^the use of by-products and plant waste to improve the quality of gluten-free food may have a varied effect on the physicochemical and sensory parameters of gluten-free products. Many researchers indicate that replacing wheat flour with various additives in the form of dried and powdered products and/or plant waste in bread production should not exceed several percentage points due to consumer assessment^47,50,51^while in the case of other cereal products, such as pasta or cookies, this level may be higher^52–54^. In the case of gluten-free bread with 5% orange pomace, the sensory evaluation was similar to the control bread^23^and the authors did not show a significant difference between the sensory characteristics of the bread, except for the “texture when chewed”. In turn, apple pomace added to gluten-free bread showed good acceptability at an 8% addition^24^. In our study, the breads without chokeberry and raspberry additions were rated the worst, while the breads enriched with these additions were rated as more tasty. In our study, the introduction of raspberry pomace to the gluten-free bread recipe had a positive effect on the improvement of all sensory properties. The only exception was the 10FD_R sample, for which the color was rated slightly worse. On the other hand, the aroma, consistency, and taste improved significantly even with a 10% share of raspberry pomace. When selecting the number of additives for bread, it is worth paying attention to the conclusions from the meta-analysis conducted by Grigor et al.^55^which showed that food products without any additives, characterized by low consumer acceptability, after enrichment with various additives, gained in acceptability. However, enriching products that already had additives decreased their acceptability. As the authors report, enrichment improved the acceptability of the texture of muffins and bread with low base acceptability, but decreased the acceptability of the texture when the base acceptability was high.

### Principal Component Analysis (PCA)

**Fig. 5:**
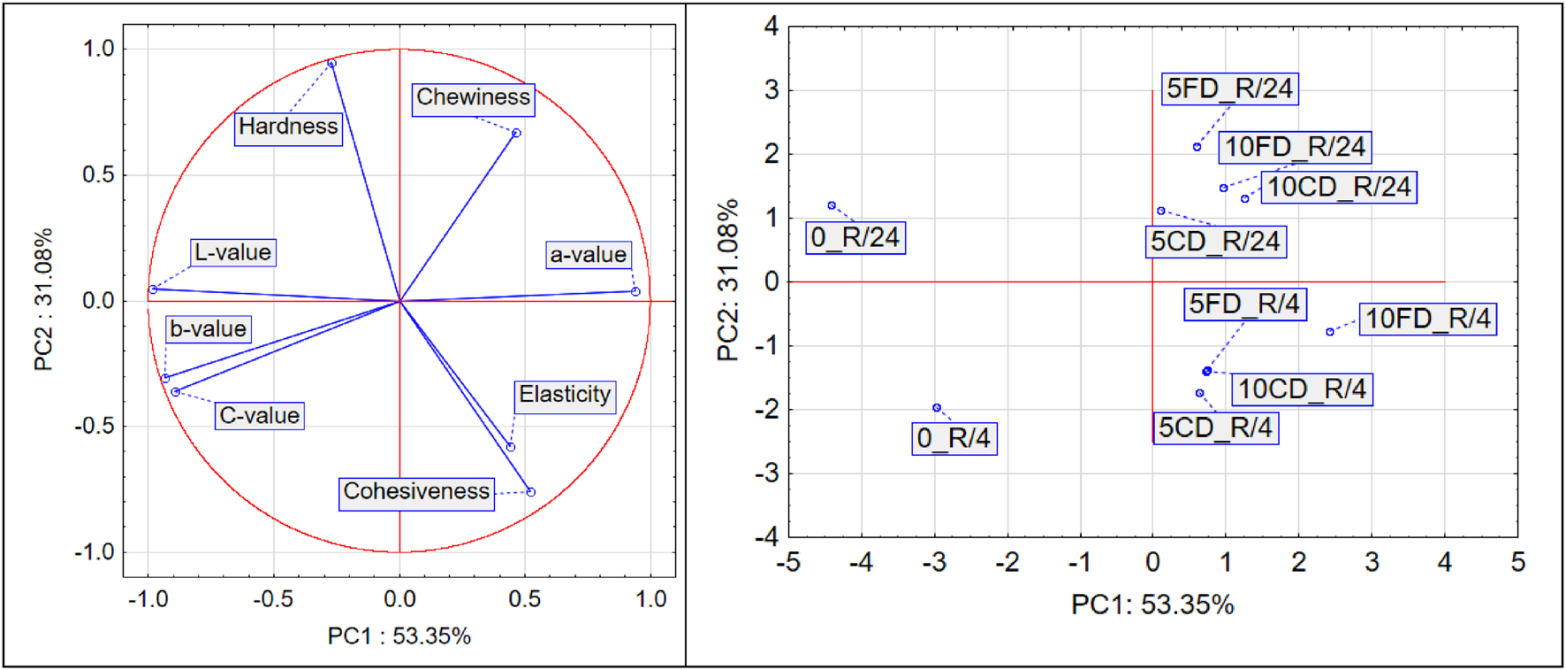
PCA of bread with raspberry pomace: (**a**) variable projection, (**b**) sample projection.

Figure 5 shows the results of PCA for breads with raspberry pomace addition.

Principal component analysis (PCA), conducted on data describing colour parameters (L*, a*, b*, C*) and textural properties (hardness, chewiness, cohesiveness, elasticity) for breads with raspberry pomace, revealed that the first two components – PC1 and PC2 – explain a total of 84.43% of the variance in the dataset (53.35% and 31.08%, respectively).

Colour parameters (L*, a*, b*, C*) strongly differentiate the samples along PC1, while textural features such as hardness, chewiness, elasticity and cohesiveness dominate PC2, indicating their independent contribution to the variance structure. The lack of significant correlation between these two groups of variables is confirmed by the nearly perpendicular arrangement of their vectors on the PCA biplot, suggesting relative independence – variability in colour does not directly correspond to changes in textural properties, and vice versa.

PCA also revealed clear relationships between the colour parameters of the tested samples. The a* value, representing red intensity, was strongly and negatively correlated with L* (lightness), b* (yellowness), and C* (chroma), which is evident from the opposing directions of their vectors on the PCA plot. This indicates that an increase in a* (more redness) is accompanied by a decrease in lightness, yellowness, and saturation. Such a relationship may suggest a shift in the dominant colour tone – from bright, yellowish tones toward darker, deeper red shades.

The PCA results also point to significant relationships among the textural attributes. Hardness and chewiness were strongly and positively correlated, as shown by the similar direction and length of their vectors. This means that samples with higher hardness also exhibited greater chewiness. In contrast, cohesiveness and elasticity formed another group of positively correlated texture parameters. However, these were negatively correlated with hardness, suggesting that an increase in sample hardness was associated with a decrease in cohesiveness and elasticity. This may be of practical relevance, as bakery products with lower hardness and higher elasticity are often better accepted by consumers.

The score plot revealed clear sample groupings. Control samples (0_R/4, 0_R/24), which did not contain raspberry pomace, were located on the left side of the plot (negative PC1 values), indicating distinct differences from the other samples. This position was associated with lower red intensity, higher lightness, and higher yellowness saturation.

The second group included samples analysed 4 h after baking, with the addition of 5% or 10% raspberry pomace, either convection dried (CD) or freeze dried (FD). These samples were located in the lower part of the plot (negative PC2), which may indicate lower hardness and chewiness immediately after baking. Their positive PC1 values, however, suggest a more intense red colour and lower brightness compared to the control group.

The third group consisted of samples analysed 24 h after baking (5CD_R/24, 10CD_R/24, 5FD_R/24, 10FD_R/24). These were located in the upper right quadrant of the PCA plot, reflecting a simultaneous enhancement of textural properties (hardness, chewiness) and maintenance of high red colour intensity (high a* values).

The PCA results thus confirm that the drying method (freeze drying vs. convection drying), raspberry pomace content, and the time of analysis significantly affect the colour and texture characteristics of the bread.

**Fig. 6:**
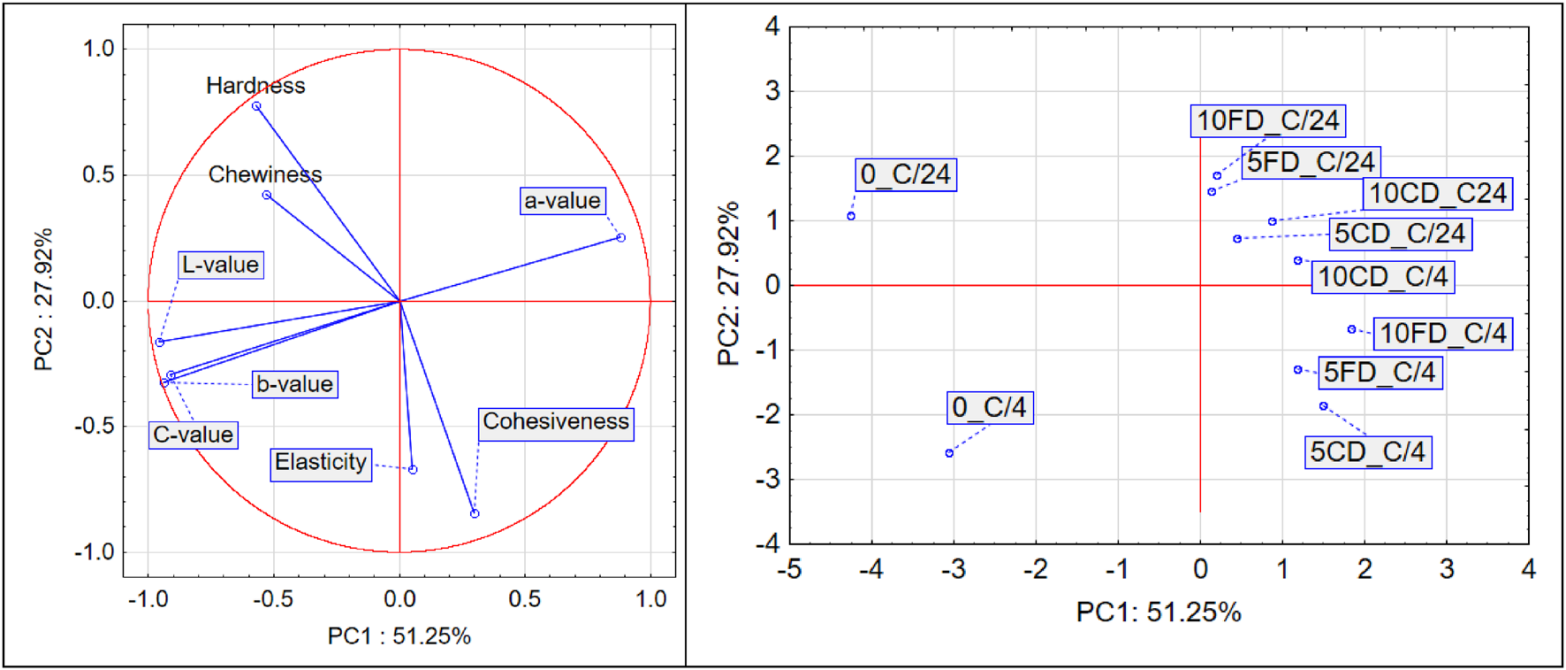
PCA of bread with chokeberry pomace: (**a**) variable projection, (**b**) sample projection.

Figure 6 presents the PCA results for breads with chokeberry pomace addition.

Principal component analysis (PCA), conducted for variables describing colour properties (L*, a*, b*, C*) and textural attributes (hardness, chewiness, cohesiveness, elasticity) in breads with chokeberry pomace addition, revealed that the first two components – PC1 and PC2 – explain a total of 79.17% of the variance in the dataset (PC1: 51.25%, PC2: 27.92%).

The first principal component (PC1) is primarily responsible for differentiating the samples based on colour characteristics such as L*, a*, b*, and C*, whereas the second component (PC2) is mainly associated with textural properties, including hardness, chewiness, cohesiveness, and elasticity. The lack of significant correlation between colour and texture was confirmed by the nearly perpendicular arrangement of the corresponding vectors on the variable projection plot, indicating that the impact of chokeberry addition on colour variability does not directly correspond to changes in textural attributes, and vice versa.

Among the colour variables, a* (redness intensity) was positively correlated with PC1 and thus with samples containing chokeberry pomace, whereas L*, b*, and C* were negatively correlated with a*. This indicates that the addition of chokeberry deepens the colour towards a darker red, while simultaneously decreasing brightness and colour saturation.

The textural attributes also formed two internal clusters: hardness and chewiness were strongly and positively correlated, as were cohesiveness and elasticity. However, these two groups were negatively correlated with each other, suggesting that an increase in hardness tends to be associated with a decrease in elasticity – a relationship also observed in the analysis of raspberry pomace addition.

The PCA score plot revealed three distinct groups of samples. Group I includes the control samples (0_C/4, 0_C/24), located on the left side of the plot (negative PC1 values). The absence of chokeberry pomace resulted in higher brightness (L*), lower red intensity (a*), and overall weaker colour. Their position also indicates relatively lower values of textural attributes (PC2).

Group II consists of samples analysed 4 h after baking. These are positioned in the lower part of the plot (negative PC2), indicating lower hardness and chewiness immediately after baking. At the same time, their clearly positive PC1 values reflect a more intense colour, particularly an increase in redness (a*) with a concurrent decrease in brightness and chroma.

Group III includes samples with chokeberry pomace analysed 24 h after baking. These are located in the upper right quadrant of the PCA plot, indicating a simultaneous increase in hardness and chewiness, along with the maintenance of high colour intensity. The most distinctive sample is 10FD_C/24, representing bread with 10% freeze dried chokeberry pomace, which reached the highest values on both principal components – indicating the most intense combined colour-texture profile among all tested samples.

The PCA results confirm that chokeberry pomace addition – particularly in freeze dried form and at a 10% concentration – significantly affects the colour and hardness of bread. These effects are further enhanced after 24 h of storage.

In the present study, we focused mainly on properties important for the consumer while we intend to develop our future experiment to include amylographic and farinographic studies, which are very important for assessing the suitability of dough for bread baking.

## Conclusion

Enriching gluten-free bread with raspberry and chokeberry pomace gave satisfactory results – despite changes in physical properties, the products were well-rated sensorially and even better received by consumers than the control bread.

The addition of pomace (convection dried and freeze dried) affected the colour parameters – the bread became darker, more red and less yellow. After 24 h, a decrease in colour intensity was observed, and the greatest colour changes were observed in the sample of bread with 10% freeze dried raspberries pomace.

The texture of the bread with raspberries did not change in terms of hardness and elasticity, although a decrease in cohesiveness was observed. The effect on chewiness was small, except for the sample with 10% freeze dried pomace raspberries. On the other hand, the addition of chokeberry pomace reduced hardness and chewiness, without affecting cohesiveness and elasticity.

It is worth emphasizing that the research we conducted indicates that the highest hardness was observed in the control sample, while the presence of fruit pomace contributed to a decrease in this parameter and thus to a delay in the reddening process of the bread.

The highest taste and smell ratings were obtained by the bread with raspberries pomace, while the colour was assessed best in the bread with chokeberry. The best was the gluten-free bread with 10% addition of freeze dried raspberries pomace, while the lowest score was given to the control bread. The highest average score was given to the sample with 5% convection dried chokeberry pomace.

The addition of pomace supports the idea of sustainable development and the use of by-products. These breads can be consumed by people with celiac disease and have the potential for functional food.

Due to the fact that higher elasticity and lower hardness are better accepted by consumers, we recommend bread with the addition of 5% convection dried chokeberry pomace (5CD_C). This bread also received the highest sum of partial scores in the sensory evaluation conducted among consumers.

To reduce the loss of bioactive compounds in gluten-free bread with raspberry or chokeberry pomace during storage, it is recommended to store the bread in cool, dark conditions with limited exposure to oxygen and light, preferably in airtight packaging or under a modified atmosphere. However, the potential for accelerated staling at lower temperatures should also be considered.

## Data Availability

All data generated or analysed during this study are included in this published article.
